# Prenatal Exposure to Acetaminophen and Childhood Asthmatic Symptoms in a Population-Based Cohort in Los Angeles, California

**DOI:** 10.3390/ijerph181910107

**Published:** 2021-09-26

**Authors:** Zeyan Liew, Yuying Yuan, Qi Meng, Ondine S. von Ehrenstein, Xin Cui, Marie E. S. Flores, Beate Ritz

**Affiliations:** 1 Department of Environmental Health Sciences, Yale School of Public Health, New Haven, CT 06510, USA; 2 Yale Center for Perinatal, Pediatric, and Environmental Epidemiology, Yale School of Public Health, New Haven, CT 06510, USA; 3 Department of Epidemiology, Fielding School of Public Health, UCLA, Los Angeles, CA 90095, USA; yuying.yn@gmail.com (Y.Y.); mengqixn@ucla.edu (Q.M.); ovehren@ucla.edu (O.S.v.E.); britz@ucla.edu (B.R.); 4 Department of Community Health Sciences, Fielding School of Public Health, UCLA, Los Angeles, CA 90095, USA; 5 Perinatal Epidemiology and Health Outcomes Research Unit, Division of Neonatology, Department of Pediatrics, School of Medicine and Lucile Packard Children’s Hospital, Stanford University, Palo Alto, CA 94304, USA; cynthiacui1010@ucla.edu; 6 California Perinatal Quality Care Collaborative, Palo Alto, CA 94305, USA; 7 Department of Health Services, Altamed, Pico Rivera, CA 90660, USA; marie.flores@utah.edu; 8 Department of Neurology, Geffen School of Medicine, UCLA, Los Angeles, CA 90095, USA

**Keywords:** acetaminophen, race/ethnicity, childhood asthma, psychosocial stress, pregnancy

## Abstract

Acetaminophen is the most common over-the-counter pain and fever medication used by pregnant women. While European studies suggest acetaminophen exposure in pregnancy could affect childhood asthma development, findings are less consistent in other populations. We evaluated whether maternal prenatal acetaminophen use is associated with childhood asthmatic symptoms (asthma diagnosis, wheeze, dry cough) in a Los Angeles cohort of 1201 singleton births. We estimated risk ratio (RR) and 95% confidence interval (CI) for childhood asthmatic outcomes according to prenatal acetaminophen exposure. Effect modification by maternal race/ethnicity and psychosocial stress during pregnancy was evaluated. The risks for asthma diagnosis (RR = 1.39, 95% CI 0.96, 2.00), wheezing (RR = 1.25, 95% CI 1.01, 1.54) and dry cough (RR =1.35, 95% CI 1.06, 1.73) were higher in children born to mothers who ever used acetaminophen during pregnancy compared with non-users. Black/African American and Asian/Pacific Islander children showed a greater than two-fold risk for asthma diagnosis and wheezing associated with the exposure. High maternal psychosocial stress also modified the exposure-outcome relationships. Acetaminophen exposure during pregnancy was associated with childhood asthmatic symptoms among vulnerable subgroups in this cohort. A larger study that assessed prenatal acetaminophen exposure with other social/environmental stressors and clinically confirmed outcomes is needed.

## 1. Introduction

Acetaminophen, also known as paracetamol, is the most common over-the-counter pain and fever medication used by pregnant women [1]. Several prospective cohort studies have reported positive associations between prenatal exposure to acetaminophen and asthma development in offspring during childhood [2,3,4]. Acetaminophen can cross the placenta, exposing the fetus during critical periods of development [5,6]. Acetaminophen was suggested to deplete glutathione (GSH) [7], a key antioxidant in the airways, and enhance immune responses that can predispose prenatally acetaminophen-exposed children to asthmatic phenotypes.

Asthma is a common non-communicable disease in childhood that was estimated to affect ~8.6% children under age 18 in the U.S. in 2014 [8]. In the California Health Interview Survey 2001–2007, asthma prevalence was higher in some ethnic minorities and families living in poverty [9]. Three meta-analyses estimated that maternal use of acetaminophen during pregnancy is associated with an overall 20–40% increased odds of developing asthma in childhood [2,3,4]. However, most evidence has come from Northern and Western European countries [10,11,12,13], and less consistent findings were reported from studies in the U.S. [14,15,16,17]. Two U.S. cohorts enrolled predominantly white and relatively wealthy families from Massachusetts or Southern New England [14,17], and two studies enrolled minority populations [15,16]. A study of 345 pregnant women (60% Mexican, 17% Puerto Rican, 11% other Hispanics, 6.4% Black/African American (AA), 6.2% other race/ethnicity) reported that maternal acetaminophen intake in middle to late but not early pregnancy was associated with respiratory symptoms in the first year of life [16]. Another study of 301 inner-city Dominican Republic and Black/AA children in New York reported that maternal use of acetaminophen in pregnancy was associated with the child’s current wheeze at age 5 [15].

We evaluated the associations between maternal use of acetaminophen during pregnancy and the offspring’s asthmatic symptoms in early childhood in a Los Angeles (LA) birth cohort. This ethnically diverse cohort allowed us to investigate potential differences in exposure-outcome associations by maternal race/ethnicity, and whether maternal prenatal psychosocial stressors [18] modify the association between acetaminophen exposure and childhood asthmatic symptoms.

## 2. Materials and Methods

### 2.1. Study Population

We analyzed maternal and child data collected from Environment and Pregnancy Outcomes Study (EPOS) at the University of California, Los Angeles (UCLA). Details of the study have been described elsewhere [19]. The source population included all singleton births from 1 January 2003 to 31 December 2003, born to mothers who resided in 111 LA County ZIP codes. The study sampling was stratified by child birth outcome characteristics and the study districts according to air pollution measures.

From a total of 58,316 eligible singleton births identified from California state and LA County electronic birth certificate records, we selected all cases of low birth weight (<2500 g) or preterm birth (<37 completed weeks gestation), and an equal number of randomly sampled controls (≥2500 g and full term) from a set of 24 ZIP codes located in close proximity to South Coast Air Quality Management District air monitoring stations. Moreover, from 87 ZIP codes that contained major population centers and were located close to major roadways, the study randomly selected 30% of all preterm or low birth weight cases and an equal number of controls. Of the 6347 women sampled, 2543 were interviewed by phone or replied to a mailed survey in English or Spanish three to six months post-partum (40% response rate). Mothers provided detailed information on pregnancy exposures and behaviors, including over-the-counter medication use.

In 2006–2007, a follow-up study was conducted with the primary aim to assess the offspring’s respiratory health. A total of 1201 (47.2% of EPOS participants) women were located again and responded to a new survey by phone or mail. The primary reason for loss to follow up was the inability to recontact women based on the initial address/phone numbers provided on birth certificates and during contact for EPOS shortly after the child was born i.e., three years prior to follow-up rather than refusal to participate. Details of the study design are described elsewhere [18]. Informed consent was obtained from the women who participated in the EPOS and the follow-up study.

### 2.2. Acetaminophen Exposure

In the baseline interviews/questionnaires conducted 3–6 months after birth, mothers were asked “Did you take any of the following medications while you were pregnant with your baby who was born in 2003?”. A list of medication was provided including the pain relievers acetaminophen, aspirin, or ibuprofen. Mothers were encouraged to specify the medication name if they did not recognize those listed in the questionnaire. If they indicated use, the mothers were asked to report the pregnancy trimester of use, defined as 1st–3rd months as first trimester, the 4th–6th months as second trimester, and 7th month till delivery as third trimester. Moreover, mothers were asked to report how often they took the medication on average during pregnancy, reporting this as once per month or less, once per week, about 3 days per week, or every day.

### 2.3. Asthmatic Outcomes

Children’s respiratory health at three to four years of age was assessed via maternal report according to the International Study of Asthma and Allergies in Childhood (ISAAC) core questionnaire [20]. The primary endpoints for this study were maternal reports of a child ever being diagnosed with asthma by a medical professional (yes/no). Moreover, mothers were asked to report a child’s history of wheezing (yes/no) or having had a dry cough at night in the past year not associated with flu or cold (yes/no) and we evaluated these as secondary endpoints.

### 2.4. Statistical Analysis

We used generalized linear models to estimate risk ratio (RR) for the three asthmatic outcomes (asthma diagnosis, wheezing, or dry cough at night) in children associated with maternal use of acetaminophen in pregnancy. We compared use of acetaminophen during pregnancy with never use as the reference. For trimester-specific analyses, we first evaluated yes/no intake in each trimester separately, and then included all three trimester-specific exposure indicators in the model simultaneously. For frequency of use during pregnancy, we classified the responses into never use (reference), once per month or less, and more than once per month. We also conducted linear trend tests for frequency of use using a cumulative index (continuous variable) that summed pre-defined scores (0 for never, 1 for once/month, 4 for once/week, and 8 for 3 days/week or more). Analyses were conducted using all mother-child pairs and then repeated within the strata of self-identified maternal race/ethnicity (White, non-Hispanic, Hispanic/Latinx, Black/AA, Asian/Pacific Islanders and others). In analyses by acetaminophen timing or frequency of use, maternal race/ethnicity other than White or Hispanic/Latinx were grouped into one category due to small sample sizes.

Confounders were selected a priori after literature review of factors possibly associated with the exposure and the outcome and based on data availability. In main analyses, we adjusted for maternal age (≤24, 25–29, 30–34, ≥35), parity (0, ≥1), household income (10–30 K, 30–50 K, >50 K), smoking (Never smoked, ever smoked but not during pregnancy, ever smoked during pregnancy), high fever during pregnancy, and maternal intake of aspirin or ibuprofen during pregnancy. Multiple imputations of 5 iterations were used to estimate the missing values of covariates based on information of the exposure and all potential confounders [21].

In sensitivity analyses, we additionally adjusted for pre-pregnancy body mass index, maternal intake of antibiotics, maternal alcohol intake during pregnancy, and air pollution. Pregnancy exposure to air-pollutants may affect pregnancy complications [22], birth outcomes [23], and childhood asthma [24] in this population. We controlled for pregnancy exposure to carbon monoxide (CO), nitrogen dioxide (NO2), and particulate pollutants (PM10 and PM2.5) generated based on maternal residential ZIP Code at the time of delivery linked to the nearest South Coast Air Quality Management District air monitoring station while accounting for distance, geography, and wind flow patterns in the basin [19].

Furthermore, we examined potential effect measure modification by child’s sex and maternal psychosocial stress during pregnancy. Boys are expected to have a higher prevalence of asthma than girls in early childhood [25]. In this cohort, high maternal psychosocial stressors during pregnancy were associated with risk of wheezing in the offspring [18]. A maternal pregnancy stress index was generated from a validated questionnaire that assessed measures of pregnancy anxiety, chronic stress, acute stress due to negative life events, and lack of paternal support [18]. We performed stratified analyses for prenatal acetaminophen use and child outcomes by high (≥75th, ≥9 points) or low/medium (<75th, <9 points) prenatal psychosocial stress. We conducted tests for heterogeneity and estimated multiplicative interaction *p*-values by entering a product-term for prenatal acetaminophen exposure and child’s sex, maternal psychosocial stress, or maternal race/ethnicity in the regression model.

Inverse probability of selection weight was used to account for potential selection bias due to non-participation at follow-up. Specifically, we modeled the probability of participation at follow-up including the exposure variable and other covariates collected in the baseline interview from all mothers. Mothers who were older, U.S.-born, white, had received more years of education, and started prenatal care early in pregnancy were more likely to be re-contacted and participated in the follow-up. Prenatal use of acetaminophen was not related to follow-up status (OR = 0.99 95% CI 0.83, 1.20). We calculated stabilized inverse-probability-weights and performed weighted regression analyses throughout. We computed 95% confidence intervals (CIs) using robust variance estimators in all weighted analyses. All statistical analyses were performed using SAS statistical software, version 9.4 (SAS Institute, Cary, NC, USA).

### 2.5. Ethical Statement

The research protocol for this study was approved by the Institutional Review Boards at UCLA (IRB#10-000617 and IRB#10-001508).

## 3. Results

The majority of women in this study self-identified as Hispanic/Latina (59.8%), half had 12 or less years of education (49.7%), and 33.6% reported an annual household income below $30,000 (Table 1). Overall, 32.7% of mothers reported ever using acetaminophen during pregnancy, 9.8% of children had received a diagnosis of asthma, and 20–25% had experienced wheezing or dry cough in early childhood.

We estimated a 25–39% higher risk for all three asthma endpoints (RR = 1.39, 95% CI 0.96, 2.00), wheezing (RR = 1.25, 95% CI 1.01, 1.54) or dry cough (RR = 1.35, 95% CI 1.06, 1.73) comparing children born to mothers who ever used acetaminophen during pregnancy with non-users, adjusting for main confounders (Table 2). Additional confounding factors did not change the effect size by more than 5% (Online Resource Table S1).

The associations between maternal acetaminophen use and childhood asthmatic symptoms for white mothers were similar to those observed for the total population, while associations in Hispanic/Latina mothers were close to null. The results were null for both U.S. and foreign-born Hispanic/Latina mothers (Online Resource Table S2). Strong associations were observed in Black/AA mothers and their children with relative risks ranging from 1.9 to 4.3 for these three asthmatic endpoints and prenatal acetaminophen exposure. More than two-fold risk increases were seen for mothers and children of Asian/Pacific Islander and other origins with prenatal acetaminophen intake.

Regarding timing of exposure, effect estimates for prenatal exposure to acetaminophen and asthmatic outcomes in children were stronger when exposure occurred in the second or the third pregnancy trimester in all women and across maternal race/ethnicity groups, except for Hispanic/Latina mothers. However, most 95% confidence intervals for gestational time specific estimates included the null because of low statistical power (Online Resource Table S3).

In terms of acetaminophen intake frequency, the risk of having an asthma diagnosis was consistently greater if acetaminophen was used more than once a month during pregnancy, compared with infrequent use (once/month or less) or never users (Table 3). The same patterns were also observed in all maternal race/ethnicity subgroups, except for Hispanic/Latina mothers and their children. However, the exposure-outcome response was not consistent for wheezing or dry cough.

**Figure 1:**
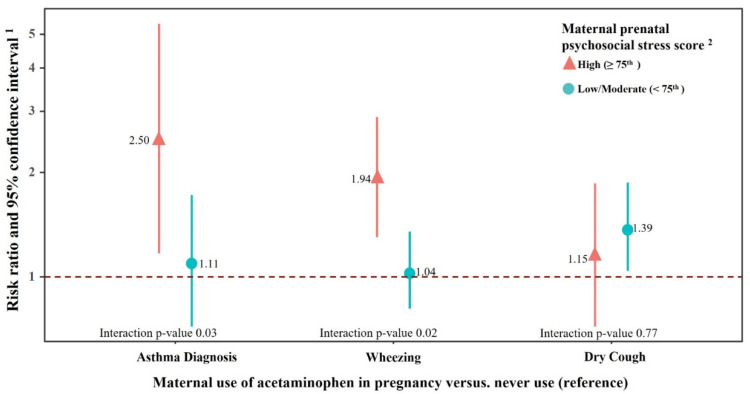
Risk ratio (RR) and 95% confidence interval (CI) for asthmatic outcomes in early childhood according to maternal prenatal acetaminophen use, stratified by maternal psychosocial stress during pregnancy. ^1^ RR was adjusted for maternal age, parity, maternal race/ethnicity, household income, smoking, maternal high fever, and aspirin or ibuprofen during pregnancy. Among women reporting high prenatal stress, 104 reported prenatal acetaminophen use, and 29, 78, and 67 children had an asthma diagnosis, wheezing and dry cough, respectively. Among women reporting low prenatal stress, 289 reported prenatal acetaminophen use, and 89, 226, and 176 children had an asthma diagnosis, wheezing and dry cough, respectively. ^2^ A maternal pregnancy stress index was generated from a validated questionnaire that assessed measures of pregnancy anxiety, chronic stress, acute stress due to negative life events, and lack of paternal support.

In analyses stratified by maternal stress, prenatal acetaminophen exposure was associated with an asthma diagnosis (RR = 2.5, 95% CI 1.17, 5.36) or wheezing (RR = 1.94, 95% CI 1.30, 2.89) among mothers who also reported high prenatal stress, but the associations were close to null for mothers with low/medium stress (interaction p-value 0.03 for asthma diagnosis and 0.02 for wheezing) (Figure 1). The effect estimates for dry cough and acetaminophen exposure largely overlapped by maternal stress strata (interaction p-value 0.77).

The effect estimates for asthma diagnosis and wheezing also appeared to be somewhat stronger among male offspring, but—as expected—fewer female offspring had asthmatic symptoms at this age, resulting in estimates with wide CIs (interaction p-value 0.34 for asthma diagnosis and 0.35 for wheezing)(Online Resource Table S4).

## 4. Discussion

In this LA birth cohort, we estimated a 25–39% higher risk for receiving an asthma diagnosis or experiencing asthmatic symptoms among children exposed to acetaminophen prenatally; these risk estimates are consistent with the most recent meta-analytic results [2,3,4]. Our study is one of few to investigate this association in a cohort of mothers and children with diverse ethnicity and socio-economic backgrounds. Some differences were observed by maternal race/ethnicity, specifically larger effect estimates for Black/AA and Asian/Pacific Islander than white children, and null results for Hispanics/Latinx children. Our study also suggested a potential modifying role of maternal psychosocial stress in pregnancy on the association between prenatal acetaminophen exposure and asthmatic outcomes in young children, which warrants further investigations.

Experimental studies suggested that acetaminophen can cause increased oxidative stress by depleting the anti-oxidant GSH, enhancing Th2 cytokine production linked to asthma, and by mediating non-eosinophilic inflammatory responses [7,26,27]. Unlike other antipyretics and analgesics, acetaminophen is not considered contra-indicated for use during pregnancy. In adults, acetaminophen is metabolized via glucuronidation, sulphation, and via the cytochrome P450 system. However, glucuronidation, the main pathway for acetaminophen metabolism in adults, is limited in the fetus [28]. Acetaminophen metabolized via the cytochrome P450 system pathway will generate a toxic metabolite called N-acetyl-p-benzoquinone imine (NAPQI). Glutathione S-transferase (GST), an essential enzyme necessary to detoxify NAPQI, can be depleted in the fetal lung with increasing exposure and in turn induces higher oxidative stress [28]. Two gene-environment interaction studies have suggested risk modifications of early life acetaminophen exposure by GST polymorphisms on asthma risk and impaired lung function in childhood [15,27].

Previous studies in our cohort indicated that maternal prenatal psychosocial stressors are associated with risk of asthmatic symptoms in childhood [18], which is consistent with findings from other epidemiologic studies [29]. The primary mechanism proposed for maternal stress is through the activation of the hypothalamic-pituitary-axis (HPA) that stimulates glucocorticoid release and increases cortisol levels; high levels of cortisol in turn potentiate cell differentiation from T helper cell type 1 (Th1) to T helper cell type 2 (Th2) phenotypes and consequently predisposes offspring to asthma [30,31]. Interestingly, prenatal stress can also affect the glutathione system, leading to a decrease in the glutathione/glutathione disulfide (GSSG) ratio and an increase in oxidative stress [30,31]. The latter mechanism is similar to the biological pathways that acetaminophen targets [7,32,33,34]. Thus, these two exposures could potentially interact to induce oxidative stress and enhance immune responses predisposing the children to the development of asthma. We explored potential synergistic effects of these prenatal exposures in our cohort and observed multiplicative interactions between acetaminophen exposure and a high maternal stress index for asthmatic outcomes in early childhood. This finding is novel and requires replication. Nonetheless, our findings highlight the importance of evaluating multiple stressors together when assessing the influence of the physical and social environment during pregnancy on the development of respiratory and asthmatic health outcomes in the offspring.

Racial/ethnic and socioeconomic disparities in the prevalence of childhood asthma have been reported in the LA county population [35]. A study of 6004 children (≤17 years old) enrolled in an LA countywide health survey between 1999 and 2000 reported that the prevalence of asthma was highest in Black/AA children (15.8%), followed by white (7.3%) and Asian children (6.0%), and lowest in Hispanics/Latinx children (3.9%) [35]. These differences in asthma prevalence persisted after controlling for household income, health care access, maternal age, number of children, and parents’ relational status. This study, however, did not evaluate whether perinatal risk factors might explain the asthma risk disparities. If maternal prenatal acetaminophen exposure is causally related to childhood asthma risk, the low prevalence and frequencies of use among Hispanic mothers could be a plausible explanation for a lower risk in this population. Moreover, a strong association found for Black/AA children suggests that prenatal medication exposures could interact with other social or environmental risk factors disproportionally affecting these families and contribute to higher asthma occurrence and symptoms. Whether acetaminophen is a causal risk factor for asthma and to what extent it explains disease racial disparities in the population needs to be further explored.

Our study has several strengths. First, the study participants were sampled from diverse LA neighborhoods. Information regarding acetaminophen use was collected shortly after birth and thus prior to asthma symptoms assessed three years later. We adjusted for a range of potential confounders, including maternal high fever and use of other pain/fever medications, and known environmental risk factors such as air pollution. Inverse-probability-weighting was applied in all models to minimize the potential influence of selection bias due to non-participation during follow-up. The proportions of prenatal acetaminophen use reported were comparable in children included or excluded in the analyses providing assurance that the exposure did not influence drop-out rates.

Several study limitations should be noted. First, acetaminophen use is common, dosage taken may vary, and mothers might not have accurately reported their use during pregnancy. Exposure misclassification may increase with length of time between acetaminophen use and answering the survey, making recall for first trimester use worse than for third trimester use; therefore, our analyses addressing exposure timing need to be interpreted with caution. However, we expect any exposure misclassification to most likely be non-differential because the exposure data was collected three years prior to outcome assessment, and acetaminophen use was considered safe in pregnancy and would not have raised concerns in health care providers during the study period. Our study adjusted for high fever and pregnancy use of other common fever and pain medications, but our analysis could not rule out residual confounding by other maternal conditions associated with intake of acetaminophen [36]. In addition, our study did not have data on postnatal acetaminophen use by children. The ISAAC is a standardized and validated instrument for assessing pediatric asthma [20], but we did not have an opportunity to confirm the outcomes clinically. Finally, symptoms such as wheezing and dry cough assessed in young children have large variability. Studies with a longer follow-up period are needed to confirm our findings.

## 5. Conclusions

In conclusion, this study provided some evidence to suggest that maternal acetaminophen use in pregnancy was associated with offspring asthmatic symptoms and wheeze in early childhood. The exposure-outcome relationship in this LA cohort was stronger for women who experienced high psychosocial stress in pregnancy and among Black/AA and Asian/Pacific Islander children. These findings would need confirmations from larger studies that assess the joint effects of acetaminophen exposure with other social and environmental stressors in pregnancy and evaluate clinically confirmed asthma in children at older ages.

## Figures and Tables

**Table 1:** Characteristics of the participants by maternal acetaminophen use during pregnancy, a population-based cohort study in Los Angeles (LA), California, 2003–2007.

Characteristics	Maternal Ever Use of Acetaminophen during Pregnancy			
	Yes (*N* = 393)		No (*N* = 808)	
	*n*	%	*n*	%
**Child’s sex**				
Male	191	48.6	418	51.7
Female	202	51.4	390	48.3
**Maternal age (years)**				
≤24	70	17.8	217	26.9
25–29	99	25.2	200	24.8
30–34	129	32.9	226	28.0
≥35	95	24.2	165	20.4
**Maternal race/ethnicity**				
White, non-Hispanic/Latina	136	34.6	162	20.0
Hispanic/Latina	203	51.7	515	63.7
Black/African American	20	5.1	51	6.3
Asian/Pacific Islanders and others ^1^	33	8.4	72	8.9
Missing	1	0.2	8	1.0
**Maternal education (years)**				
<12	70	17.8	250	30.9
12	89	22.6	188	23.3
13–15	77	19.6	136	16.8
≥16	151	38.4	215	26.6
Missing	6	1.5	19	2.4
**Parity**				
Nulliparous	154	39.2	337	41.7
Parous	239	60.8	471	58.3
**Household income (dollars)**				
10–30 K	104	26.5	299	37.0
30–50 K	72	18.3	164	20.3
>50 K	178	45.3	245	30.3
Missing	39	9.9	100	12.4
**Mother’s U.S. born status**				
U.S. born	221	56.2	348	43.1
Non-U.S. born	170	43.3	460	56.9
Missing	2	0.5	0	0
**Maternal smoking**				
Ever smoked during pregnancy	22	5.6	31	3.8
Ever smoked but not during pregnancy	134	34.1	215	26.6
Never smoked	237	60.3	562	69.6
**Maternal BMI**				
<18.5	15	3.8	35	4.3
18.5–24.9	202	51.4	391	48.4
25–29	74	18.8	174	21.5
>29	70	17.8	116	14.4
Missing	32	8.1	92	11.4
**Maternal alcohol intake during pregnancy (yes)**	53	13.5	76	9.4
Missing	1	0.3	0	0
**Mother high fever during pregnancy (yes)**	125	31.9	186	23.0
Missing	1	0.3	1	0.1
**Maternal antibiotic use during pregnancy** **(yes)**	14	3.6	34	4.2
**Maternal aspirin use during pregnancy (yes)**	25	6.4	32	4.0
**Maternal ibuprofen use during pregnancy**	19	4.8	22	2.7

^1^ Others include American Indian, Indian (excluding American Indian, Eskimo and Aleut), Filipino, Hawaiian, Guamanian, Samoan, Eskimo, Aleut, and other specified races.

**Table 2:** Risk ratio (RR) and 95% confidence interval (CI) for asthmatic outcomes in early childhood according to maternal prenatal acetaminophen use, stratified by maternal self-identified race/ethnicity.

Maternal Race/Ethnicity	Acetaminophen Use during Pregnancy	No. of All Children	Asthma Diagnosis			Wheezing			Dry Cough		
			*N*	RR ^1^ (95% CI)	Interaction *p*-Value ^1^	*N*	RR ^1^ (95% CI)	Interaction *p*-Value ^1^	*N*	RR ^1^ (95% CI)	Interaction *p*-Value ^1^
Total population	Never	808	71	Ref		186	Ref		147	Ref	
	Ever	393	47	1.39 (0.96, 2.00)	n/a	118	1.25 (1.01, 1.54)	n/a	96	1.35 (1.06, 1.73)	n/a
White, not Hispanic	Never	162	9	Ref		35	Ref		29	Ref	
	Ever	136	14	1.52 (0.68, 3.40)	0.15	44	1.27 (0.86, 1.87)	0.36	32	1.15 (0.74, 1.80)	0.97
Hispanic/Latina	Never	515	50	Ref		118	Ref		93	Ref	
	Ever	203	17	0.79 (0.45, 1.40)	Ref	50	1.05 (0.78, 1.42)	Ref	42	1.17 (0.82, 1.68)	Ref
Black/African American	Never	51	6	Ref		19	Ref		12	Ref	
	Ever	20	9	4.27 (1.93, 9.46)	<0.01	12	2.04 (1.23, 3.40)	0.05	10	1.88 (0.93, 3.77)	0.12
Asian/Pacific Islander and others ^2^	Never	72	6	Ref		12	Ref		12	Ref	
	Ever	33	7	2.55 (0.91, 7.16)	0.08	11	2.18 (1.08, 4.40)	0.23	12	2.66 (1.26, 5.63)	0.16

^1^ Adjusted for maternal age, parity, maternal race/ethnicity, household income, smoking, maternal high fever, and aspirin or ibuprofen during pregnancy among total population; Adjusted for all except for maternal race/ethnicity in analyses by maternal race/ethnicity subgroups. ^2^ Others include American Indian, Indian (excluding American Indian, Eskimo and Aleut), Filipino, Hawaiian, Guamanian, Samoan, Eskimo, Aleut, and other specified races.

**Table 3:** Risk ratio (RR) and 95% confidence interval (CI) for asthmatic outcomes in early childhood according to the frequency of maternal prenatal acetaminophen use.

Maternal Race/Ethnicity	Acetaminophen Use during Pregnancy	No. of All Children	Asthma Diagnosis			Wheezing			Dry Cough		
			*N*	RR ^1^ (95% CI)	*p*-Trend ^2^	*N*	RR ^1^ (95% CI)	*p*-Trend ^2^	*N*	RR ^1^ (95% CI)	*p*-Trend ^2^
Total population	Never use	808	71	Ref		186	Ref		147	Ref	
	Once a month or less	231	27	1.41 (0.90, 2.20)		74	1.37 (1.08, 1.74)		61	1.47 (1.11, 1.95)	
	More than once a month	136	19	1.56 (0.96, 2.52)	0.07	39	1.18 (0.87, 1.60)	0.18	29	1.17 (0.81, 1.70)	0.39
White, not Hispanic	Never use	162	9	Ref		35	Ref		29	Ref	
	Once a month or less	86	8	1.41 (0.62, 3.22)		28	1.27 (0.84, 1.93)		20	1.10 (0.67, 1.82)	
	More than once a month	40	5	1.67 (0.52, 5.34)	0.07	14	1.32 (0.74, 2.33)	0.06	9	1.13 (0.57, 2.23)	0.63
Hispanic/Latina	Never use	515	50	Ref		118	Ref		93	Ref	
	Once a month or less	115	10	0.86 (0.43, 1.72)		32	1.24 (0.88, 1.75)		28	1.37 (0.92, 2.04)	
	More than once a month	76	7	0.80 (0.37, 1.76)	0.41	16	0.83 (0.51, 1.36)	0.38	13	0.92 (0.52, 1.63)	0.56
Others ^3^	Never use	123	12	Ref		31	Ref		24	Ref	
	Once a month or less	29	9	3.54 (1.56, 8.03)		13	2.18 (1.32, 3.57)		13	2.61 (1.47, 4.66)	
	More than once a month	20	7	5.00 (2.33, 10.71)	<0.001	9	2.00 (1.20, 3.33)	<0.001	7	1.89 (0.96, 3.71)	<0.001

^1^ Adjusted for maternal age, parity, maternal race/ethnicity, household income, smoking, maternal high fever, and aspirin or ibuprofen during pregnancy among total population; Adjusted for all except for maternal race/ethnicity in analyses by maternal race/ethnicity subgroups. ^2^ A cumulative exposure index modeled continuously that summing pre-defined scores of 0 for never use, 1 for once/month, 4 for once/week, and 8 for 3 days/week or more. ^3^ Includes Black/African American, Asian/Pacific Islander, American Indian, Indian (excluding American Indian, Eskimo and Aleut), Filipino, Hawaiian, Guamanian, Samoan, Eskimo, Aleut, and other specified races.

## Data Availability

The data is being privately stored to protect the anonymity of the participants.

## Supplementary Materials

The following are available online at https://www.mdpi.com/article/10.3390/ijerph181910107/s1, Table S1: Risk ratio (RR) and 95% confidence interval (CI) for asthmatic outcomes in early childhood according to maternal prenatal acetaminophen use, Table S2: Risk ratio (RR) and 95% confidence interval (CI) for asthmatic outcomes in early childhood according to maternal prenatal acetaminophen use among Hispanic/Latinx, stratified by maternal birth place, Table S3: Risk ratio (RR) and 95% confidence interval (CI) for asthmatic outcomes in early childhood according to maternal acetaminophen use in each pregnancy trimester, Table S4: Risk ratio (RR) and 95% confidence interval (CI) for asthmatic outcomes in early childhood according to maternal prenatal acetaminophen use, stratified by child’s sex.
