# Supplementary material for: Prenatal Exposure to Acetaminophen and Childhood Asthmatic Symptoms in a Population-Based Cohort in Los Angeles, California
Source: Int J Environ Res Public Health. 2021 Sep 26;18(19):10107. doi: 10.3390/ijerph181910107 (PMC8507827; doi:10.3390/ijerph181910107)
Supplement: Supplementary file 1 [file ijerph-18-10107-s001.zip › ijerph-1370157-supplementary/Supplementary tables final.pdf]

Title: Prenatal exposure to acetaminophen and childhood asthmatic symptoms in a population-based cohort in Los Angeles, California.

Authors and Affiliations Zeyan Liew, PhD, MPH; Yuying Yuan, MPH; Qi Meng, MS; Ondine S. von Ehrenstein, PhD; Xin Cui, MD, PhD; Marie E.S. Flores, MD, PhD, MPH; Beate Ritz, MD, PhD

Correspondence Address correspondence to Zeyan Liew, Department of Environmental Health Sciences, Yale School of Public Health. Full address: *60 College Street, New Haven CT* 06510. Email: zeyan.liew@yale.edu

Table S1. Risk ratio (RR) and 95% confidence interval (CI) for asthmatic outcomes in early childhood according to maternal prenatal acetaminophen use.

	Never use during pregnancy (N=808)	Ever use during pregnancy (N=393)	RR (95% CI)		
			**Model A** [a]	**Model B** [b]	**Model C** [c]
Asthma diagnosis	71	47	1.39 (0.96, 2.00)	1.35 (0.93, 1.95)	1.38 (0.96, 1.98)
Wheezing ever	186	118	1.25 (1.01, 1.54)	1.22 (0.99, 1.51)	1.25 (1.01, 1.54)
Dry cough ever	147	96	1.35 (1.06, 1.73)	1.32 (1.03, 1.69)	1.35 (1.05, 1.73)

a. Model A: adjusted for maternal age, parity, maternal race/ethnicity, household income, smoking, maternal high fever, and aspirin or ibuprofen during pregnancy.
b. Model B: adjusted for all in Model A and pre-pregnancy BMI, use of antibiotics, maternal alcohol intake during pregnancy.
c. Model C: adjusted for all in Model A and exposures to carbon monoxide (CO), nitrogen dioxide (NO_2), PM10 and PM2.5 during pregnancy.

Table S2. Risk ratio (RR) and 95% confidence interval (CI) for asthmatic outcomes in early childhood according to maternal prenatal acetaminophen use among Hispanic/Latinx, stratified by maternal birthplace.

	Acetaminophen use during pregnancy	No. of all children	Asthma diagnosis		Wheezing		Dry cough	
			N	RR [a] (95% CI)	N	RR [a] (95% CI)	N	RR [a] (95% CI)
**U.S born Hispanic/Latinx**	Never	154	16	Ref	46	Ref	27	Ref
	Ever	74	9	0.95 (0.42, 2.16)	21	0.96 (0.60, 1.53)	16	1.18 (0.63, 2.24)
**Foreign born Hispanic/Latinx**	Never	361	34	Ref	72	Ref	66	Ref
	Ever	129	8	0.68 (0.30, 1.54)	29	1.11 (0.75, 1.66)	26	1.17 (0.76, 1.82)

a. Adjusted for maternal age, parity, household income, smoking, maternal high fever, and aspirin or ibuprofen during pregnancy.

Table S3. Risk ratio (RR) and 95% confidence interval (CI) for asthmatic outcomes in early childhood according to maternal acetaminophen use in each pregnancy trimester.

		First trimester exposure				Second trimester exposure				Third trimester exposure			
		**No**	**Yes**	**RR [a] (95%CI)**	**RR [b] (95% CI)**	**No**	**Yes**	**RR [a] (95% CI)**	**RR [b] (95% CI)**	**No**	**Yes**	**RR [a] (95% CI)**	**RR [b] (95% CI)**
**Total population**	Asthma diagnosis	103	15	0.82 (0.48, 1.41)	0.61 (0.31, 1.20)	88	30	1.33 (0.88, 2.02)	1.42 (0.84, 2.38)	91	27	1.25 (0.82, 1.91)	1.25 (0.74, 2.12)
	Wheezing	249	55	1.19 (0.90, 1.56)	1.10 (0.79, 1.54)	232	72	1.11 (0.87, 1.41)	0.97 (0.71, 1.33)	231	73	1.23 (0.97, 1.56)	1.20 (0.90, 1.62)
	Dry cough	203	40	1.13 (0.81, 1.57)	0.91 (0.61, 1.34)	179	64	1.34 (1.02, 1.75)	1.26 (0.90, 1.76)	183	60	1.32 (1.00, 1.74)	1.22 (0.87, 1.71)
**White, not Hispanic**	Asthma diagnosis	18	5	0.70 (0.26, 1.85)	0.48 (0.12, 1.93)	13	10	1.20 (0.55, 2.63)	1.33 (0.41, 4.39)	14	9	1.23 (0.53, 2.84)	1.40 (0.42, 4.61)
	Wheezing	54	25	0.99 (0.64, 1.52)	0.86 (0.50, 1.48)	48	31	1.08 (0.73, 1.60)	0.97 (0.51, 1.86)	48	31	1.18 (0.79, 1.77)	1.30 (0.70, 2.43)
	Dry cough	44	17	0.84 (0.51, 1.37)	0.66 (0.35, 1.24)	37	24	1.14 (0.73, 1.79)	1.27 (0.66, 2.45)	39	22	1.12 (0.70, 1.79)	1.18 (0.61, 2.30)
**Hispanic/Latinx**	Asthma diagnosis	62	5	0.50 (0.20, 1.23)	0.53 (0.19, 1.47)	56	11	0.89 (0.45, 1.75)	1.17 (0.57, 2.42)	59	8	0.64 (0.30, 1.35)	0.70 (0.31, 1.58)
	Wheezing	146	22	1.34 (0.90, 1.97)	1.41 (0.91, 2.19)	139	29	1.00 (0.69, 1.46)	0.89 (0.58, 1.36)	140	28	1.05 (0.73, 1.52)	1.00 (0.66, 1.51)
	Dry cough	120	15	1.20 (0.72, 1.99)	1.16 (0.65, 2.08)	111	24	1.02 (0.66, 1.59)	0.88 (0.53, 1.46)	110	25	1.25 (0.83, 1.89)	1.26 (0.81, 1.97)
**Others [c]**	Asthma diagnosis	23	5	1.87 (0.79, 4.42)	0.86 (0.24, 3.06)	19	9	2.76 (1.36, 5.58)	2.00 (0.76, 5.26)	18	10	2.93 (1.44, 5.98)	2.33 (0.83, 6.50)
	Wheezing	47	7	1.24 (0.65, 2.35)	0.82 (0.35, 1.91)	42	12	1.42 (0.85, 2.39)	1.14 (0.61, 2.13)	40	14	1.88 (1.18, 2.99)	1.91 (1.07, 3.41)
	Dry cough	38	8	1.49 (0.76, 2.95)	0.86 (0.40, 1.88)	30	16	2.71 (1.68, 4.35)	2.44 (1.51, 3.93)	33	13	2.03 (1.16, 3.55)	1.49 (0.78, 2.85)

a. Model A: adjusted for maternal age, parity, maternal race/ethnicity, household income, smoking, maternal high fever, and aspirin or ibuprofen during pregnancy.

b. Model B: all in Model A and mutually adjusted for first, second, and third trimester exposure in the same model.

c. Includes Black/African American, Asian/Pacific Islander, American Indian, Indian (excluding American Indian, Eskimo and Aleut), Filipino, Hawaiian, Guamanian, Samoan, Eskimo, Aleut, and other specified races.

Table S4. Risk ratio (RR) and 95% confidence interval (CI) for asthmatic outcomes in early childhood according to maternal prenatal acetaminophen use, stratified by child's sex.

		Acetaminophen use during pregnancy			
	Child's sex	Never use (N)	Ever use (N)	RR [a] (95% CI)	Interaction p-value [a]
**Asthma diagnosis**	Male	43	33	1.59 (1.03, 2.45)	ref
	Female	28	14	1.08 (0.55, 2.12)	0.34
**Wheezing**	Male	110	74	1.35 (1.04, 1.75)	ref
	Female	76	44	1.13 (0.79, 1.60)	0.35
**Dry cough**	Male	87	53	1.24 (0.90, 1.71)	ref
	Female	60	43	1.48 (1.01, 2.15)	0.75

a. Adjusted for maternal age, parity, maternal race/ethnicity, household income, smoking, maternal high fever, and aspirin or ibuprofen during pregnancy.
